# Achieving Procedural Parity in Managing Access to Genomic and Related Health Data: A Global Survey of Data Access Committee Members

**DOI:** 10.1089/bio.2022.0205

**Published:** 2024-04-15

**Authors:** Jonathan Lawson, Vasiliki Rahimzadeh, Jinyoung Baek, Edward S. Dove

**Affiliations:** ^1^ Data Sciences Platform, Broad Institute of MIT and Harvard, Cambridge, Massachusetts, USA.; ^2^ Center for Medical Ethics and Health Policy, Baylor College of Medicine, Houston, Texas, USA.; ^3^ School of Law, University of Edinburgh, Edinburgh, United Kingdom.

**Keywords:** data access, data sharing, data management, data use

## Abstract

Data access committees (DACs) are critical players in the data sharing ecosystem. DACs review requests for access to data held in one or more repositories and where specific constraints determine how the data may be used and by whom. Our team surveyed DAC members affiliated with genomic data repositories worldwide to understand standard processes and procedures, operational metrics, bottlenecks, and efficiencies, as well as their perspectives on possible improvements to quality review. We found that DAC operations and systemic issues were common across repositories globally. In general, DAC members endeavored to achieve an appropriate balance of review efficiency, quality, and compliance. Our results suggest a similarly proportionate path forward that helps DACs pursue mutual improvements to efficiency and compliance without sacrificing review quality.

## Introduction

Compliant and timely access to genomic and related health data is consequential for both genomic science and ethics. Researchers require access to data to drive scientific discovery and innovation. At the same time, rights and interests of individuals and communities must be respected in the process of such data discovery and innovation. Data access committees (DACs) are critical players in the data sharing ecosystem.^1^ DACs review requests for access to data held in one or more repositories and where specific constraints determine how the data may be used and by whom.^2^

**FIG. 1.**
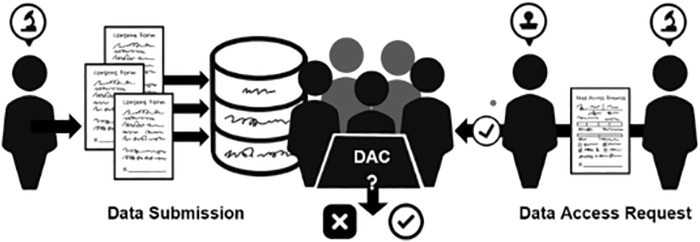
Prototypical workflow and primary duties of a DAC. DAC, data access committee.

DACs may consist of one or more members with various expertise levels who oversee multiple distinct datasets or repositories.^3^ DAC members may also liaise with researchers within, as well as external to, institutions, sectors, and countries.^4^ Through this role, they are responsible for verifying that only authorized users are granted access to potentially sensitive data, such as genomic data, for certain approved purposes (Fig. 1).^5^

DAC roles and responsibilities are largely conserved across databases and knowledge repositories around the world,^3^ yet only recently have standardized procedures been developed to guide efficient, consistent, and fair DAC review.^6^ Unstandardized procedures preclude evaluation of DAC review processes and outcomes needed to monitor stewardship of valuable data resources.^7^

Moreover, current approaches to DAC review are based on static approaches to genomic data generation and largely rely on manual workflows to manage data access requests (DARs).^3^ In increasingly dynamic cloud and quantum computing environments, such manual approaches can result in procedural inconsistencies and delays in access for authorized researchers^8^ and can complicate compliance monitoring.^9^

New research also suggests that DAC review directly impacts study quality and scientific rigor.^10^ For example, results from a qualitative study of genetics researchers found that ease of access motivates their decisions to use datasets from private companies rather than government or other publicly funded datasets, which tend to require lengthy data access review.^11^

Datasets from companies such as direct-to-consumer genetic testing companies may, however, lack genetic diversity since they contain data shared by consumers and are not necessarily representative of all populations intended to benefit from genomic science.^12^

Ensuring compliant and efficient access to data creates a clear opportunity for consensus development of robust global standards for both manual and automated decision support for DAC review.^13^ We broadly refer to automated decision support systems as rule-based systems that analyze data inputs and apply algorithmic logic to aid in decisional outputs.

Through standardization, DACs may instill greater confidence that primary and secondary research data use is upheld over the research data's lifecycle, while concurrently pruning administrative redundancies. We believe that data access review may soon become a rate-limiting step in genomic data discovery^2^ as demand for complex datasets grows in the absence of standards guiding how DACs can operate more consistently.

Despite their critical roles in managing compliant access to genomic and related health data, DACs are vastly understudied groups of institutional stakeholders. Standardization of DAC procedures has therefore been difficult to achieve thus far because empirical data on internal DAC operations, workflows, structures, and review outcomes are severely limited.

To fill this knowledge gap, we conducted an anonymous survey of DAC members affiliated with genomic data repositories around the world. We report on the results from this global survey and the implications they raise for developing standardized procedures to guide efficient, consistent, and fair DAC review for access to genomic and related health data moving forward.

## Methods

Prospective participants were recruited from a volunteer working group of DAC professionals led by the authors, V.R., J.L., and E.S.D., known as the Data Access Committee Review Standards Working Group (DACReS WG). The DACReS WG is a special interest group within the Global Alliance for Genomics and Health (GA4GH). The GA4GH is a nonprofit organization formed in 2013 that aims to accelerate progress in genomic research and human health by cultivating a common framework of standards and harmonized approaches for effective and responsible genomic and health-related data sharing.

Additional survey respondents were recruited following a systematic internet search of active, publicly funded genomic data repositories based in the United States (e.g., affiliated with a university, research institution, health/hospital system) as well as internationally. Follow-up inquiries were sent to administrative DAC offices where specific contact information was unavailable. Links to participate in the survey were sent to each identified contact to ensure maximum geographical and professional variation in the sample.

The survey length, item order, and language were further refined following three separate pilot studies with individuals familiar with genomic data access and management. The administered survey is provided in the Supplementary Material. Only members of DACs affiliated with genomic data repositories were recruited to complete our survey as guided by our research objective to understand the current procedures, workflows, and quality perceptions of DACs managing genomic and related health data.

This study was deemed exempt by the Stanford University Institutional Review Board.

## Results

The survey took ∼15 minutes to complete and comprised the following sections of questions: demographics and institutional background, DAC operations and workflow, and perceptions of automated workflow solutions. A total of 35 participants completed the survey between March 2 and June 30, 2022. We proceed to highlight the main results below.

### Demographics and institutional background

Of the 35 participants, 51% were female and 46% were male. Most participants (94%) were over 30 years of age, and most held graduate/postgraduate and/or professional degrees. Fifty-two percent served on the DAC for more than a year, and 29% served over 5 years (Table 1).

**Table 1.** Demographics and Institutional Background

Characteristics	Total (*n* = 35) % (*n*)
Gender	
Female	51 (18)
Male	46 (16)
Age, years	
18–29	6 (2)
30–45	49 (17)
45–60	37 (13)
60+	9 (3)
Highest degree earned	
Undergraduate	6 (2)
Graduate/postgraduate	69 (24)
Professional	23 (8)
How long have you served on the DAC?	
<1 Year	18 (6)
1–2 Years	26 (9)
3–4 Years	26 (9)
Over 5 years	29 (10)
Location of DACs	
North America	34 (12)
Europe	34 (12)
Australia, New Zealand, or Pacific Island nations	14 (5)
Asia	9 (3)
Africa	6 (2)
South America	3 (1)
Type of institution	
Government research agency	31 (11)
Nonprofit research institute	28 (10)
Academic affiliated research institute	17 (6)
Consortium	17 (6)
Health care/hospital system	6 (2)
Corporate, commercial, or other for-profit institution	3 (1)

DAC, data access committee.

DAC members represented research institutions based in North America (34%), Europe (34%), Australia (14%), Asia (8%), Africa (6%), and South America (3%). These institutions included government research agencies (31%), nonprofit research institutions (29%), academic research institutions (17%), consortia (17%), health systems (3%), and for-profit entities (3%) (Table 2).

**Table 2.** Types and Numbers of Datasets

Characteristics	Total (*n* = 35) % (*n*)
Types of datasets	
Genomes	83 (29)
Exomes	71 (25)
Single cell	34 (12)
Human samples	63 (22)
Imaging data	20 (7)
Number of datasets under management	
<50	29 (10)
50–500	35 (12)
Over 500	35 (12)

Across these institutions, DACs were predominantly responsible for managing access to genomic data of different types (genomes 83%, exomes 71%, and single cell data 34%)—which is to be expected given the sensitivity of genomic data and various policy constraints conveying the need for DAC-like oversight.

We found that 63% of the DAC members surveyed were also responsible for managing access to human samples, in addition to genomic data listed above, and an additional 20% were responsible for managing access to imaging data. Of DACs responding to the survey, 35% managed over 500 datasets, 29% managed <50 datasets, and 35% managed between 50 and 500 datasets.

### Internal DAC operations: problems of variation

The survey data indicate that DAC structure, membership, and decision-making vary widely. While little-to-no direct legislation or policy governing DACs exists globally, most institutions established their own standard operating procedures (SOPs) (82%) and terms of reference (63%) to guide DAC operations. Three primary rationales motivated the development of SOPs, including a need for consistency and efficiency in the DAC review process (80%), to foster transparency and accountability (74%), and to achieve objective decision-making (54%).

Seventy-one percent of respondents reported that DAC service was not formally included in their job descriptions. Of the DAC members who were formally appointed to serve on a DAC (29%), more than half came from government research agencies, and only 6% spent a majority of their time on DAC-related work (Table 3).

**Table 3.** Data Access Committee Operations

Survey questions	Total (*n* = 35) % (*n*)
Does your DAC have terms of reference?	
Yes	63 (22)
No	34 (12)
I don't know	3 (1)
Does your DAC have established SOPs?	
Yes	82 (27)
No	18 (6)
What prompted your DAC to develop SOPs?	
Need for consistency and efficiency in the review process	80 (28)
To foster transparency and accountability	74 (26)
Need for objective decision-making	54 (19)
How many members serve on your DAC?	
2–4	35 (11)
5–10	45 (14)
10+	19 (6)
Is DAC service an established role in your job description?	
Yes	29 (10)
No	71 (25)

SOPs, standard operating procedures.

#### DAC workflow: problems of resource constraints

Human-only workflows for overseeing compliant data access and use are both time and resource intensive. They are also unlikely to scale to meet the exponential growth in requests for genomic data without significant increases in costs.^14^ We therefore surveyed DAC members about how their committee currently processes DARs. We found that 11% of DACs allow a single member to process DARs, while 52% of DACs assign processing to a subset of DAC members (e.g., two or three members).

Thirty-seven percent of DACs require a full committee to process DARs, which often means that more than five members review the DAR separately and reach a consensus decision following committee deliberation. Moreover, DACs apply different methods for making final decisions about a DAR. For example, 41% of DACs require unanimous consensus. Other DACs (41%) require a majority vote, while 15% rely on the DAC chair to issue a final decision, often considering the prior comments and/or votes of the DAC members.

The volume and complexity of DARs also varied significantly. Respondents reported that 57% of DACs they serve received <10 DARs per month. Nearly 26% of DACs received between 10 and 100 DARs per month, while 17% received more than 100 DARs. While reported burdens were least for DACs with under 10 DARs per month (57%), 71% of DAC members reported that DAC service was not explicitly outlined in their job description.

As such, we believe it is reasonable to assume that DAC members with no allocated effort reviewing 10 DARs per month will process DARs less efficiently than those with a 50%–100% allocation of effort to DAC work, who process ≥100 DARs per month (Table 4).

**Table 4.** Data Access Committee Workflow

Survey questions	Total (*n* = 35) % (*n*)
Who participates in reviewing DARs?	
Full committee	37 (13)
Subset of committee members	52 (18)
Single member	11 (4)
How does your DAC make a final decision on a DAR?	
Majority vote	41 (13)
Unanimous consensus	41 (13)
The committee chair ultimately decides	15 (5)
Individual committee members make a final decision	3 (1)
On average, how many new DARs does your DAC receive per month?	
<10	57 (20)
11–100	26 (9)
100+	17 (6)
On average, how many DARs does your DAC review per month?	
<10	65 (22)
11–100	29 (10)
100+	6 (2)

DARs, data access requests.

Researchers, DACs, and data sharing enthusiasts^10^ share a desire to expedite data access. While more than a third (36%) of respondents attested that they issue decisions on DARs to researchers within 1 week, 58% of DACs required a minimum of 2 weeks to process a DAR and 27% of DACs took at least 1 month. Based on these projections, a researcher requesting data from four different DACs for co-analysis is likely to be delayed by at least 1 month before gaining access to the data they need.

When questioned about the cause(s) for delays, many participants attributed them to missing data/incomplete information in the DAR form submitted by the researcher (71%). Participants additionally noted that “verifying a researcher's identity” (34%), dealing with “different interpretations of data sharing terms…among the DAC” (29%), and “verifying an institution's legitimacy” (23%) were among the key reasons for delayed access decisions.

A deeper dive into DACs' interpretation of consented data uses showed that 63% of DACs “often” or “always” review original/underlying consent forms when reviewing DARs (Table 5).

**Table 5.** Data Access Committee Decision Process

Survey questions	Total (*n* = 35) % (*n*)
On average, how long does it take for your DAC to complete its review of a DAR?	
Less than a week	36 (12)
1 Week	6 (2)
2–3 Weeks	30 (10)
4–5 Weeks	21 (7)
More than 6 weeks	6 (2)
What are the most common causes of review delays?	
Missing data/incomplete information in the DAR submitted by the researcher	71 (25)
Verifying researcher's identity and bona fides	34 (12)
Different interpretations of data sharing terms/allowable data uses across members of DAC	29 (10)
Verifying institution's legitimacy/trustworthiness	23 (8)
How often does your DAC consult the original consent form when assessing the data use terms of a new access request?	
Always	19 (6)
Often	44 (14)
Rarely	25 (8)
Never	12 (4)

#### Broad interest and healthy skepticism in automated approaches to DAC review and approval

In light of the demands on DACs and relevant standards and software solutions designed to facilitate data sharing, we examined DAC perceptions of applying automated decision support tools to improve DAC workflows. We provided details of the GA4GH Data Use Ontology (DUO)^15^ as one example of an automated decision support tool.

The GA4GH DUO provides a shared understanding of the meaning of data use categories and is distributed as a machine-readable file that encodes both how the data can be used (data use categories) and how a researcher intends to use the data (additional terms that define intended research usage).^16^

We surveyed DAC members about the perceived benefits and limitations of standardizing data use permissions using a tool such as the GA4GH DUO.^15^ DAC members reported that some of the perceived benefits included reducing the processing time (43%), ensuring consistency in the review process (43%), having clarity among DAC members on the data use terms of the dataset (41%), improving interoperability (31%), and reducing the member workload (29%).

When asked about using software to facilitate DAC workflows, participants identified that record keeping and auditability (86%) were notable perceived benefits, as well as ensuring consistency in the review process (71%), reducing the processing time (63%), and improving interoperability (54%). Nine percent of respondents noted no perceived benefits to software support.

In a follow-up, participants were asked about potential challenges to the software-facilitated DAC workflow. Participants expressed some skepticism. They were concerned about the technical complexity of implementing software tools (49%) and that promises of efficiency gains were unclear (29%) and various issues involving robust data security were unclear (26%). Ultimately, those DAC members interested in moving to the software-facilitated workflow noted the largest perceived benefits as efficiency gains (46%), along with consistency in the review process (21%) (Table 6).

**Table 6.** Perceptions of Software

Survey questions	Total (*n* = 35) % (*n*)
What are the benefits of having permitted uses for your datasets in a standardized format?	
Reducing the processing time	43 (15)
Ensuring consistency in the review process and the results	43 (15)
Having a clear shared understanding of the meaning of the data use categories (minimizes misunderstanding not only among members of the DAC but also between the DAC and requesters)	41 (14)
Improving interoperability	31 (11)
Reducing member workload	29 (10)
What are the potential benefits of using software to conduct data access reviews?	
Record-keeping/auditability	86 (30)
Ensuring consistency in the review process	71 (25)
Reducing the processing time	63 (22)
Improving interoperability	54 (19)
There are no benefits	9 (3)
What are the potential challenges of using software to conduct data access reviews?	
Technically too complicated, difficult, or annoying to use/learn	49 (14)
Efficiency gains are insignificant or nonexistent	29 (10)
Security concerns	26 (9)
Why are you interested in using software to conduct data access review?	
Efficiency gains	46 (16)
Consistency in the review process	21 (7)
Fairness	9 (3)

With the potential for semiautomation of DAR decisions in an advanced search algorithm system such as DUOS (the Broad Institute's Data Use Oversight System), which leverages the DUO, we surveyed participant interest in automating DARs based on the level of permissibility of use of the dataset.

Of those participants who reported interest in automating some elements of the DAR review process, 46% wanted more information before proceeding, and 60% supported automating DARs based on specific DUO profiles (i.e., GRU, HMB, and DS-tagged datasets). On the other hand, 23% expressed little to no interest in pursuing DUOS now or in the future (Table 7).

**Table 7.** Perceptions of Automation

Survey questions	Total (*n* = 35) % (*n*)
Would you be interested in using a system that automatically approves/rejects DARs without human review for the following data use types?	
Interested, but unsure of the technicalities of such a system	46 (16)
General research use	29 (10)
Broad consent with no restrictions	26 (9)
Health/medical/biomedical research use	17 (6)
Disease-specific research use	14 (5)
Population origins/ancestry research	11 (4)
Not at all interested	23 (8)

## Discussion

We hypothesized that demand for data would likely decline over time as datasets aged, leading to a decrease in overall DARs. However, almost no DAC members we surveyed experienced a decline in demand. Rather, 50% of DAC members reported an increase in DARs over time, and 41% reported DAR demand as steady.

We consider one possible reason for this relationship. As prior research has shown, greater availability of genomic data, coupled with more researchers with necessary data analysis tools and skill sets, leads to increases in demand for genomic data and DARs.^13^ In other words, the demand increase from the growing research community is outpacing the otherwise diminishing interest in individual datasets, buoying the work required of DACs.

We thus surmise that the need for efficient DAC workflows will only increase over time, although some of the delays may be attributable to lack of allocated DAC staff. One U.S. example testifies to this problem. National Institutes of Health (NIH)/NCBI officials who oversee the dbGaP system and manage tens of thousands of DARs annually have recently noted that they “cannot simply be sped up by throwing more people at the crank to turn it faster”.^17^

### Interest in software-supported DAC workflow trends with DAR volume and complexity

With the projected exponential increase in demand and inability to match the growth rate by simply hiring more people, streamlining processes while maintaining procedural compliance is a must to catalyze the full potential of biomedical big data and advance genomics.

Based on our experience and discussions with DAC members, expediting the DAR review process seems to be most readily achieved by (1) standardizing and validating DAR form inputs and (2) establishing efficient ways to identify an authorized researcher and institution. GA4GH colleagues, including the authors of this article, have contributed to the GA4GH Passport Standard^5^ to aid in researcher authentication and authorization from trusted sources through federated mechanisms.

While software and other automated decision support tools are attuned to expediting new or existing standard processes and procedures consistently, they are one of a myriad solutions for improving the efficiency of DAC work while strengthening compliance measures throughout. The strengths of software include the ability to configure system settings to accommodate unique data protection policies and DAC review processes where and when they undergo reform.

Software could also help DACs reach a higher potential for efficiency and compliance, as we have argued elsewhere,^18^ when facilitated by procedural standardization. DACs would theoretically be freed from developing access review criteria and policies *ad hoc*, allowing DACs to adapt software bespoke to the global standard(s) and facilitating the federation of data access for researchers, irrespective of institutional type or place.

In turn, DACs could reduce the burden of effort and wait time on researchers seeking access to data by having fewer (or at least more interoperable) systems, providing access to many/most DACs and the data they manage.

We note from DAC members with both positive and negative views of software-enabled standardization that compliance is a major concern. DAC software provides one possible solution to improve upon key compliance and ethical concerns in the DAC process, although questions remain regarding the complexity and logistics of implementation, as well as uncertainty about efficiency gains and data security.

We anticipate that procedural standardization and consistent application of review criteria, facilitated by software and automated decision support tools, are benefits that could be generalizable to DAC review for access to non-genomic datasets of various other health data types as well. For example, leveraging the GA4GH DUO in DAC decision-making bases all DARs on logically equivalent grounds, bringing equity to the process, and further tracking of the software system makes it easy to audit the consistency of DAC decisions.^19^

Furthermore, the standardization of application forms across DACs, whether through software or purely in PDF, allows under-resourced DACs to preserve consistency and efficiency and benefit from lessons learned of higher-throughput DACs.

## Conclusions

Our survey of global DAC members affiliated with genomic data repositories indicates strong support for the procedural benefits of standardization and the pressing need for efficient, consistent, and fair data access review. Survey respondents expressed general support for software-enabled opportunities to reliably reduce DAR processing time and DAC member workload, but they perceived their technical implementation to be too complex to yield measurable benefit at the onset of adoption.

Several respondents raised caution about the overreliance on automated decision tools in the DAC review process and worried that such tools would eliminate opportunities for human oversight and ethical reflection. Additional research with DAC members and other international data stewards is needed to further nuance the implementation barriers and facilitators of decision support tools across the secondary use and access pipeline.

Genomic data science is a global venture, and genomic data governance must similarly be attuned to ethical norms and practices on a global scale if it is to protect the rights and informational welfare of all data contributors equitably. The publicly funded nature of many DACs accentuates the need for access review policies and practices that are equity driven.

Engaging primarily with publicly funded DACs in this study aligns with the goals of bottom-up stakeholder engagement and, if successful, the DACReS WG plans to scale up these standardization efforts to include private and other nonpublicly funded DACs in the future.

## Supplementary Material

Supplemental data
