# Supplementary material for: Achieving Procedural Parity in Managing Access to Genomic and Related Health Data: A Global Survey of Data Access Committee Members
Source: Biopreserv Biobank. 2024 Apr 15;22(2):123–9. doi: 10.1089/bio.2022.0205 (PMC11265613; doi:10.1089/bio.2022.0205)
Supplement: Supplemental data [file bio.2022.0205_suppl_data.pdf]

Glossary of terms

Data access committee. A body of one or more named individuals who are responsible for reviewing applications for data access and use and authorizing the data's release.
Data access request. A formal or informal application for permission to access restricted data for research or other named purposes.
Data use limitation/restriction. A written description of limitations on the distribution and use of human data submitted to a controlled-access repository.
Data use agreement. A contractual document used for the transfer of non-public or restricted use data.
Data Use Ontology (DUO). A hierarchical vocabulary of human and machine-readable data use terms.
Data use term. The conditions under which non-public or restricted data may be used.
Terms of reference. Formal rules that dictate the purposes and structures for people serving on the DAC e.g., term limits, eligibility criteria, etc.
Standard operating procedure. Step-by-step instructions compiled by the institution to help members carry out routine DAC operations.

Part 1 (Demographics)

Part 1 of the survey will ask optional demographic questions about you.

1. **How old are you?**
   - ☐ 18-29
   - ☐ 30-45
   - ☐ 45-60
   - ☐ Over 60
2. **What is your gender? Select all that apply.**
   - ☐ Female
   - ☐ Male
   - ☐ Non-binary
   - ☐ Transgender
   - ☐ Intersex
   - ☐ Let me type…
   - ☐ I prefer not to say
3. **Which category best describes you? Select all that apply.**
   - ☐ White (e.g., German, Irish, English, Italian, Polish, French, etc)
   - ☐ Hispanic, Latino or Spanish origin (e.g., Mexican or Mexican American, Puerto Rican, Cuban, Salvadoran, Dominican, Colombian, etc)
   - ☐ Black or African American (e.g., African American, Jamaican, Haitian, Nigerian, Ethiopian, Somalian, etc)
   - ☐ Asian (e.g., Chinese, Filipino, Asian Indian, Vietnamese, Korean, Japanese,

etc)
- ☐ American Indian or Alaska Native(e.g., Navajo nation, Blackfeet tribe, Mayan, Aztec, Native Village or Barrow Inupiat Traditional Government, Nome Eskimo Community, etc)
- ☐ Middle Eastern or North African (e.g., Lebanese, Iranian, Egyptian, Syrian, Moroccan, Algerian, etc)
- ☐ Native Hawaiian or Other Pacific Islander (e.g., Native Hawaiian, Samoan, Chamorro, Tongan, Fijian, etc)
- ☐ Some other race, ethnicity or origin
- ☐ I prefer not to say

4. What is your highest degree earned?
- ☐ Undergraduate (e.g., BS/BSc/BA)
- ☐ Graduate/post-graduate (e.g., MA/MS/MSc/PhD)
- ☐ Professional (e.g., MD/JD/MBBS)
- ☐ I prefer not to say

Part 2 (background information)

Part 2 of the survey will ask general questions about your institution's DAC and its data management activities.

5. Where is your institution located?
- ☐ North America
- ☐ South America
- ☐ Europe
- ☐ Africa
- ☐ Asia
- ☐ Australia, New Zealand or Pacific Island nations

Country ________________
Province/State _____________

6. What is your institution type?
- ☐ Government research agency (e.g., National Institutes of Health)
- ☐ Academic affiliated research institute (e.g., the Broad Institute of MIT and Harvard)
- ☐ Non-profit research institute (i.e., affiliated with neither government nor academic institution)
- ☐ Corporate, commercial, or other for-profit institution (e.g., Pfizer)
- ☐ Healthcare/hospital system
- ☐ Consortium (e.g., Psychiatric Genomics Consortium)
- ☐ Other (e.g., single study/research group) ____________________

7. **What type(s) of datasets does your institution store and/or manage? Select all that apply.**
   - ☐ Genomes
   - ☐ Exomes
   - ☐ Single cells
   - ☐ Human samples
   - ☐ Other ____________

8. **How many datasets are under your institution's management?**
   - ☐ Less than 50
   - ☐ 50 to 99
   - ☐ 100 to 499
   - ☐ 500 to 999
   - ☐ 1,000 to 4,999
   - ☐ Over 5,000

9. **On average, how many new data access requests (DARs) does your Data Access Committee (DAC) <u>receive</u> per month?**
   - ☐ Less than 50
   - ☐ 50 to 99
   - ☐ 100 to 499
   - ☐ 500 to 999
   - ☐ Over 1,000

10. **On average, of these new DARs, how many are from requesters affiliated with foreign/international institutions?**
    - ☐ Less than 20%
    - ☐ 21 to 40%
    - ☐ 41 to 60%
    - ☐ 61 to 80%
    - ☐ 81 to 100%

Part 3 (DAC-specific questions)

Part 3 of this survey will ask general questions about the members of your DAC and your roles/responsibilities.

11. **Which of the following best describes the type of DAC at your institution?**
    - ☐ Appointed members of a standing committee
    - ☐ Volunteer members of an standing committee

- ☐ Volunteer members of an informal committee
- ☐ Single-member, researcher/principal investigator (the data producer) (go to Q13)
- ☐ Other ____________________

12. How many members are in your DAC?
- ☐ 1
- ☐ 2 - 4
- ☐ 5 - 7
- ☐ 8 - 10
- ☐ More than 10

13. How long have you served on the DAC?
- ☐ Less than a year
- ☐ 1 to 2 years
- ☐ 3 to 4 years
- ☐ Over 5 years

14. Is DAC service an established role in your job description (i.e., as part of your employment agreement or otherwise)?
- ☐ Yes (go to 14.1)
- ☐ No (go to Q15)

14.1. If yes, what percent effort (i.e. as a percentage of your working time) are you expected to dedicate to DAC service based on your job description?

____

14.2. What percent effort do you actually dedicate to DAC service in practice?

____

15. What is the average term of service of the members of your DAC?
- ☐ Less than a year
- ☐ 1 to 3 years
- ☐ 3 to 5 years
- ☐ Over 5 years
- ☐ I don't know

16. Which of the following best describes DAC meetings at your institution?
- ☐ Regularly scheduled
- ☐ Ad hoc or as needed
- ☐ Both regularly scheduled and ad hoc/as needed
- ☐ Other ____________________

17. On average, how frequently does your DAC meet to review data access requests?
- ☐ Weekly
- ☐ Biweekly

- ☐ Monthly
- ☐ Quarterly
- ☐ Other ___________________

18. Which of the following, if any, serve as DAC members? Select all that apply

- ☐ Data producers
- ☐ Research participants
- ☐ Patient advocates
- ☐ Community/local advisory board members
- ☐ None

Part 4 (DAC-specific questions) continued

Part 4 of this survey will ask general questions about the internal operations of your DAC.

19. Does your DAC have terms of reference (i.e., formal rules determining member constitution, e.g., term limits, eligibility criteria, etc.)?

- ☐ Yes
- ☐ No
- ☐ I don't know

20. Does the DAC have established Standard Operating Procedures (SOPs) (e.g., review protocols, voting rules, etc.)?

- ☐ Yes (go to Q20.2)
- ☐ No
- ☐ I don't know (go to Q 21)

20.1. If not, is there an initiative to develop SOPs in the near future?

- ☐ Yes
- ☐ No
- ☐ I don't know

20.2. If your DAC has or is developing SOPs, what prompted this? Select all that apply.

- ☐ Increased data access requests
- ☐ Increased datasets available
- ☐ Need for consistency and efficiency in review process
- ☐ Need for objective decision-making
- ☐ To foster transparency and accountability
- ☐ Other ____________
- ☐ I don't know

21. On average, how many DARs does the DAC <u>review</u> per month?

- ☐ 1 to 10
- ☐ 11 to 20
- ☐ 21 to 50

- ☐ 51 to 100
- ☐ More than 100

22. Have you perceived a change in the number of DARs received during your service on a DAC?

- ☐ Yes, perceived increase
- ☐ Yes, perceived decrease
- ☐ No, DARs have more or less stayed the same
- ☐ I don't know

23. Which of the following is available to data requesters on your website? Select all that apply.

- ☐ Assessment (access and refusal) criteria
- ☐ Number of data access requests received
- ☐ Information on approved requests
- ☐ Information on declined requests
- ☐ Data security incidents
- ☐ Data Access Agreement (A standard Data Access Agreement)
- ☐ Standardized form for data access request application
- ☐ Other information ______________________
- ☐ *We do NOT make any of this information available*

24. If you provide a standard data access agreement, is it negotiable?

- ☐ Yes
- ☐ No
- ☐ I don't know

24.1. Are the terms in your data access agreement negotiable if the data access requesting party requests changes to one or more of the terms?

- ☐ Yes
- ☐ No
- ☐ I don't know

24.2. Do the terms of your data access agreement change based on the requested datasets available within your institution?

- ☐ Yes
- ☐ No
- ☐ I don't know

Part 5 (DAC workflows)

Part 5 of the survey will ask general questions about how your DAC conducts its reviews and makes decisions about requests for data access.

25. In an average week, to your knowledge, how many hours does the DAC spend reviewing DARs?

- ☐ Less than 5 hours

- ☐ 6 to 10 hours
- ☐ 11 to 20 hours
- ☐ 21 to 30 hours
- ☐ More than 30 hours

26. On average, how long does it take your DAC from the moment of receipt of DAR submission to DAR decision?

- ☐ Less than a week
- ☐ 1 week
- ☐ 2 to 3 weeks
- ☐ 4 to 5 weeks
- ☐ More than 6 weeks
- ☐ I don’t know

27. How does the DAC primarily review DARs?

- ☐ As a full committee
- ☐ A subset of a full committee (e.g., 2 or more members or assigned reviewer(s))
- ☐ Individually
- ☐ Other____________

28. In what ways does your DAC conduct its work?

- ☐ Meeting-based, in-person or virtual (e.g., Zoom)
- ☐ Digital correspondence (e.g., email, chat platform such as Slack, Teams)
- ☐ Software application/tools

29. How does your DAC make a final decision on DARs?

- ☐ Majority vote
- ☐ Unanimous consensus
- ☐ DAC Chair ultimately decides
- ☐ Other ___________________
- ☐ I don’t know

30. What are the causes, if any, for variation in your DAC’s data access review processes? Select all that apply.

- ☐ Little to no variation occurs between DARs
- ☐ Lack of Standard Operating Procedures
- ☐ Varying degrees of expertise across DACs
- ☐ Conflicts of interest
- ☐ A variety of datasets requires different processes, perspectives, criteria, etc.
- ☐ Lack of a standardized data access request form
- ☐ Other ________________________________
- ☐ I don’t know

31. How confident are you that a DAR reviewed by two DACs (e.g., your DAC and one other) would result in a similar access decision?

- ☐ Very confident

- ☐ Somewhat confident
- ☐ Not at all confident
- ☐ I don't know

32. What are the most common causes of delays for reviews? Select all that apply.

- ☐ Ambiguous data sharing language in the original (underlying) consent form
- ☐ Different interpretations of data sharing terms/allowable data uses among members of the DAC
- ☐ Verifying researcher's identity and bona fides
- ☐ Verifying institution's legitimacy/trustworthiness
- ☐ Missing data/incomplete information in the DAR submitted by researcher
- ☐ Other ________________________

33. When reviewing a DAR (either as a group or individually), how much time on average do you spend confirming the researchers' proposed uses comply with the datasets' use terms/limitations?

- ☐ Less than 25% of the time
- ☐ 25- 50% of the time
- ☐ 50-75% of the time
- ☐ More than 75% of the time

34. What software, if any, does your DAC currently use to receive, review, and store information on DARs?

- ☐ dbGaP
- ☐ EGA
- ☐ eDAM
- ☐ DUOS
- ☐ Other ____________________

34.1 What are the potential benefits you would desire if your DAC were to use software to facilitate its work? Select all that apply.

- ☐ Save expenses
- ☐ Reduce processing time
- ☐ Ensure consistency in review process
- ☐ Improve interoperability
- ☐ Record-keeping/auditability
- ☐ Provide a second-opinion on our DAR decisions
- ☐ I see no benefits
- ☐ Other _______________________________

34.2 If you would NOT be interested in such matching system software, what are the reasons? Select all that apply.

- ☐ Technically too complicated, difficult or annoying to use/learn

- ☐ Concerned about security
- ☐ Concerned system will be inaccurate
- ☐ Efficiency gains are insignificant or non-existent
- ☐ All DAC work should be conducted by humans alone, without any software or digital support
- ☐ Other ____

35. How often do you find that researchers' proposed uses of data, described in DARs are ambiguous or difficult to evaluate against consented data use terms?

- ☐ Always
- ☐ Most of the time
- ☐ Sometimes
- ☐ Never

36. How helpful, if at all, would it be for DACs and data requesters to have a shared understanding of the meanings of data use/sharing terms?

- ☐ Very helpful
- ☐ Somewhat helpful
- ☐ Somewhat unhelpful
- ☐ Very unhelpful

37. When reviewing a DAR, does the DAC typically look back at the original consent form to ensure the request is in line with the consent form?

- ☐ Yes (go to section A)
- ☐ No (go to section B)

Section A

A37.1. How much time does the DAC spend, *per DAR,* interpreting data use/sharing) terms in the consent form?

- ☐ Less than 25% of the time
- ☐ 25- 50% of the time
- ☐ 50-75% of the time
- ☐ More than 75% of the time

A37.2. What benefits, if any, are there in having permitted uses for your datasets in a standardized format? Select all that apply.

- ☐ Reduce processing time
- ☐ Free up DAC's workload
- ☐ Have a clear, shared understanding of the meaning of the data use categories (minimize misunderstanding among the DAC members, but also between DAC and requesters)
- ☐ Ensure consistency in the review process and the results
- ☐ Improve interoperability
- ☐ Other ________________________________
- ☐ I see no benefits

Section B

B37.1. If no, does your DAC have a method or system (e.g., annotations, indexes, catalogs for your datasets) for mapping data use permissions expressed in consent forms into a standardized format?

- ☐ Yes
- ☐ No
- ☐ I don't know

B.37.1.1.If yes, could you describe the method or system?

- ☐ GA4GH Data Use Ontology (DUO)
- ☐ NIH Data Use Limitations (DULs)
- ☐ Other

____

(please briefly describe who produced your current system)

Part 6 (DUO)

Part 6 of the survey will ask general questions about your DAC's current or desired use of software to facilitate DAC review.

NOTE: DUO is distributed as a computer-readable file. Using a standardized, computer-readable vocabulary for data use like DUO helps enable DACs to (fully or partially) automate data access review.

38. **How interested would you be in using a system that checks the compatibility between data use terms (per the consent form) and intended uses (described in the DARs) and displays the results to the DAC?**
    - ☐ Very interested
    - ☐ Somewhat interested
    - ☐ Neutral
    - ☐ Not at all interested

39. **How interested would you be in using a system that leverages the DUO to automatically approve/reject DARs, even if only for a subset of data use limitations (i.e. broad consent with no restrictions, general research use)?**
    - ☐ Very interested
    - ☐ Somewhat interested
    - ☐ Neutral
    - ☐ Not at all interested

    39.1. Why would you be interested?

    ____

    39.2. Why would you not be interested?

    ____

40. **Would you be interested in participating in a 30 min follow-up interview (conducted virtually between June to September 2022)? If yes, provide us with the best email address to contact you.**

    ____
